# Supplementary material for: Invasive alien pests threaten the carbon stored in Europe’s forests
Source: Nat Commun. 2018 Apr 24;9:1626. doi: 10.1038/s41467-018-04096-w (PMC5915461; doi:10.1038/s41467-018-04096-w)
Supplement: Supplementary file 1 — Supplementary Information [file 41467_2018_4096_MOESM1_ESM.pdf]

Supplementary Material

Invasive alien pests threaten the carbon stored in Europe's forests

Rupert Seidl, Günther Klonner, Werner Rammer, Franz Essl, Adam Moreno, Mathias Neumann, Stefan Dullinger

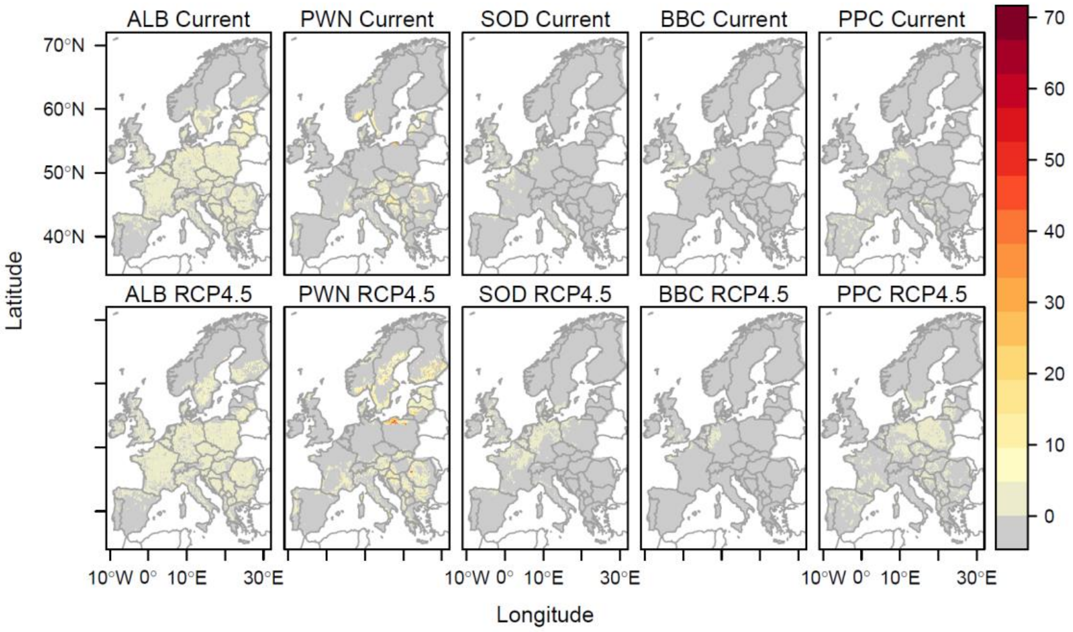

Supplementary Figure 1: Spatial distribution of live tree C at risk from invasive alien pests under current climate (top row, years 1950 – 2000) and under intermediate climate change (scenario RCP4.5, bottom row, years 2030 – 2080). Values are in Mg C ha^{-1}. See Table 1 for species abbreviations.

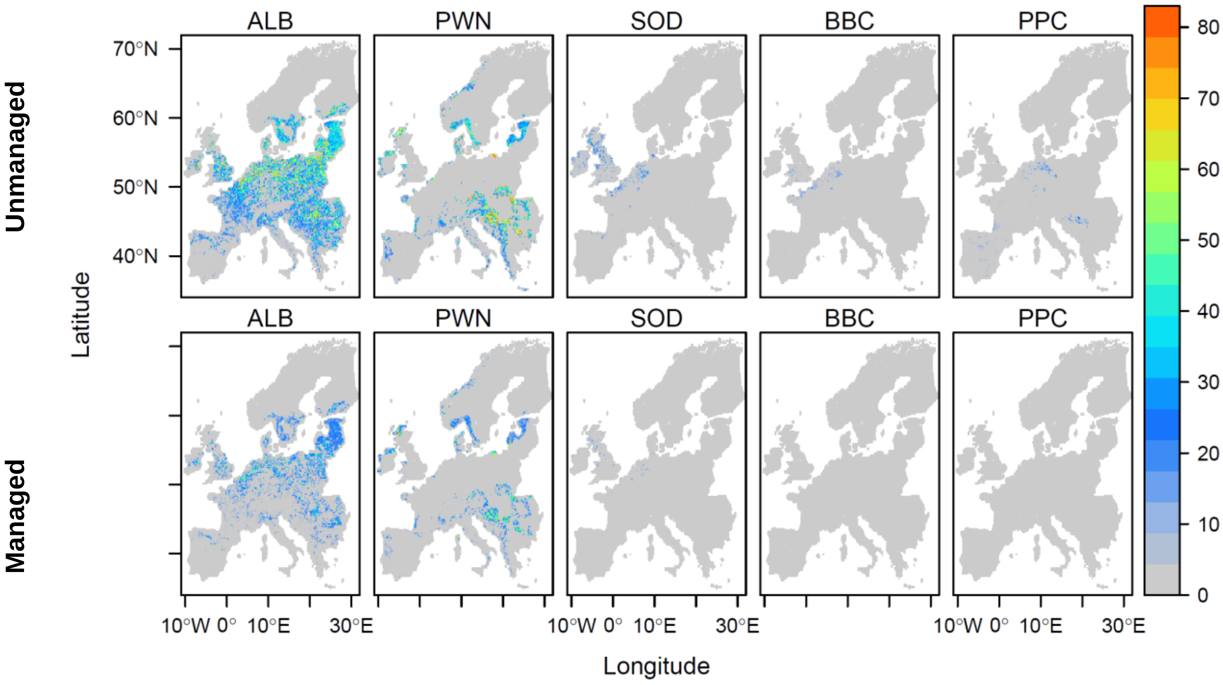

Supplementary Figure 2: Spatial distribution of carbon recovery time (years) without (top row) and with (bottom row) pest management under current climate (1950 – 2000). See Table 1 for species abbreviations.

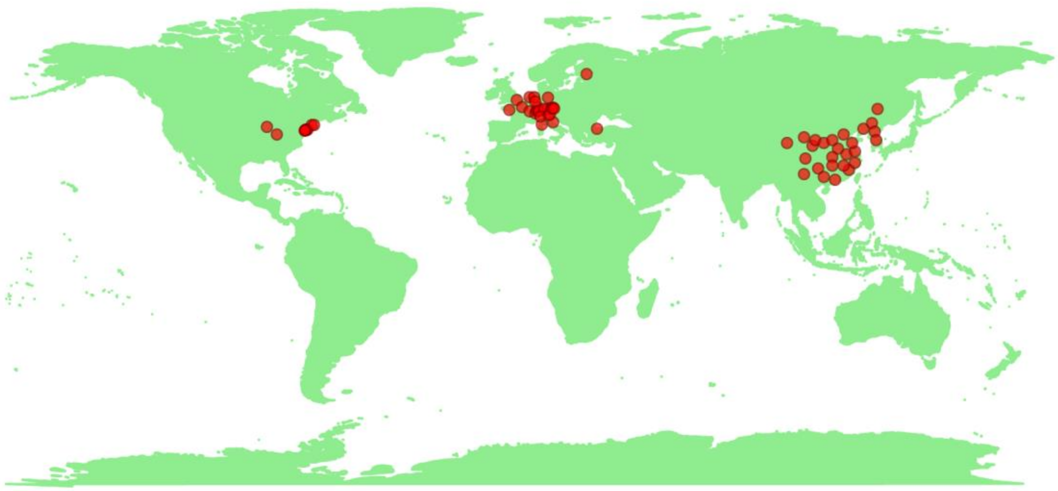

Supplementary Figure 3: Observed occurrences of the Asian Long-horned Beetle (ALB, *Anoplophora glabripennis*). Sources: [1–5].

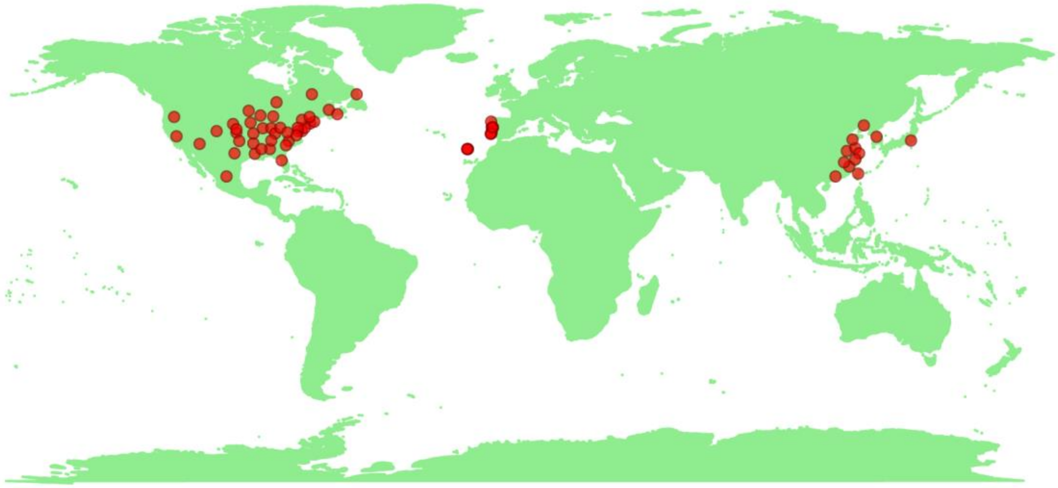

Supplementary Figure 4: Observed occurrences of the Pine Wood Nematode (PWN, *Bursaphelenchus xylophilus*). Sources:[2,6].

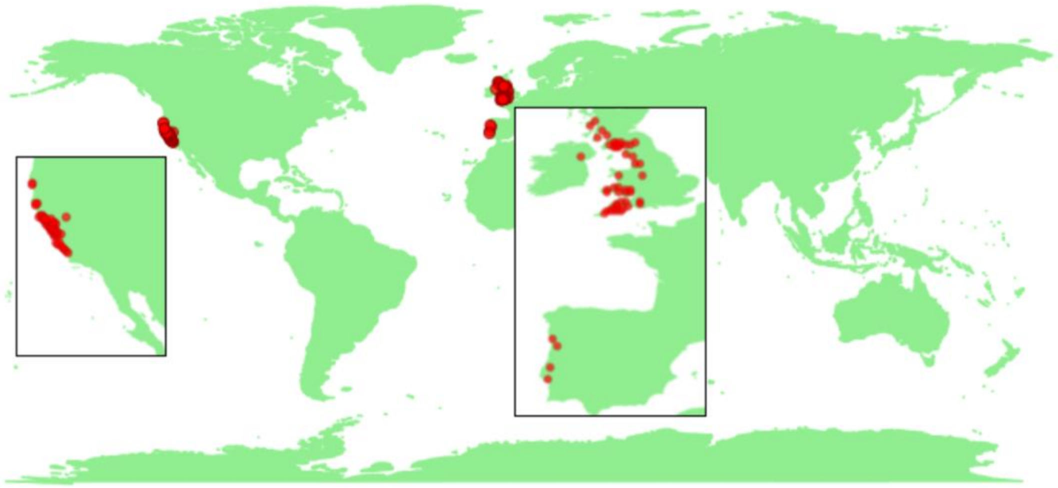

Supplementary Figure 5: Observed occurrences of Sudden Oak Death (SOD, *Phytophthora ramorum*). Sources: [2,7–11].

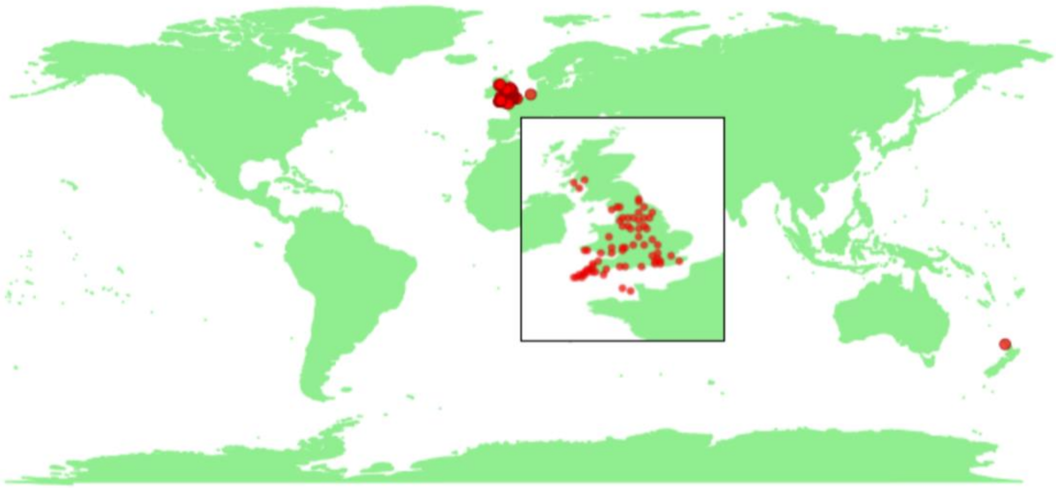

Supplementary Figure 6: Observed occurrences of Beech Bleeding Canker (BBC, *Phytophthora kernoviae*). Sources: [2,12].

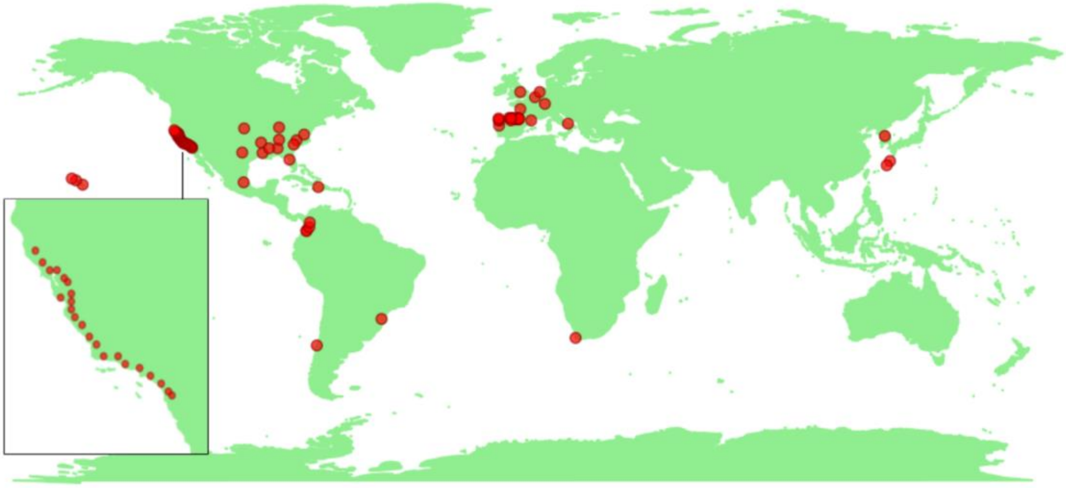

Supplementary Figure 7: Observed occurrences of Pitch Pine Canker (PPC, *Fusarium circinatum*). Sources: [2,8,13–19].

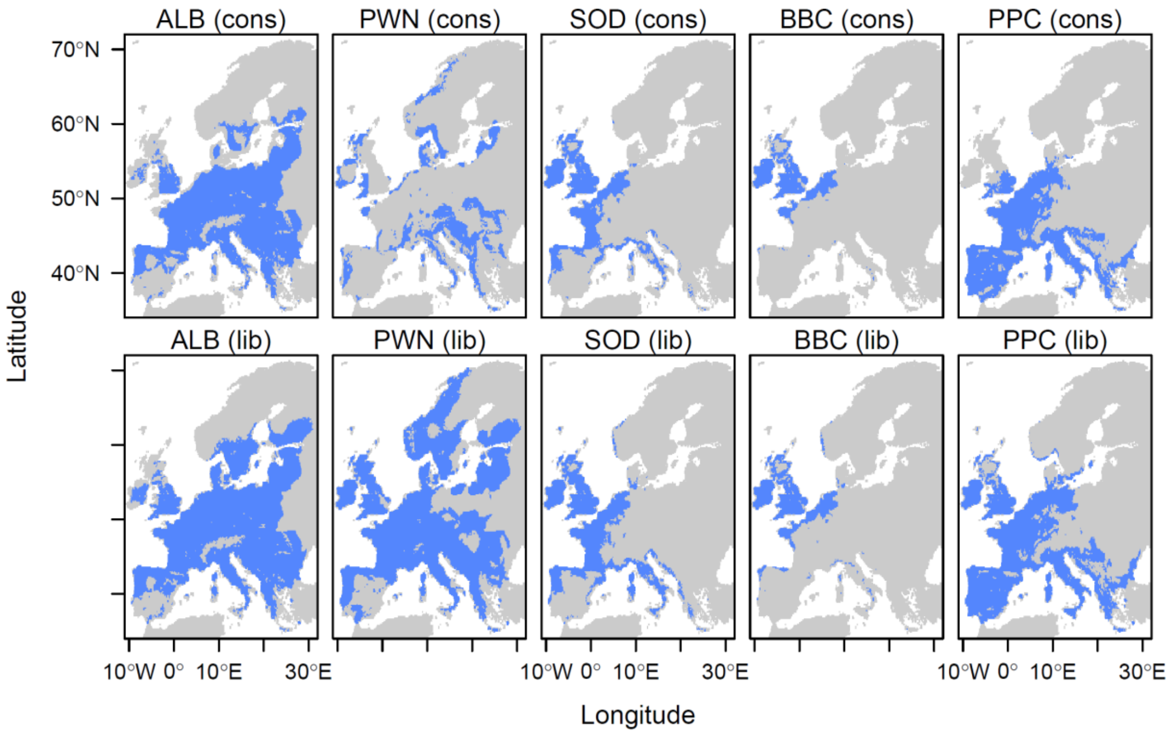

Supplementary Figure 8: Climatically suitable area (blue) for five forest pests under current climatic conditions (1950 – 2000) and different assumptions of model aggregation. Top row: consensus prediction; bottom row: liberal model aggregation. See Table 1 for species details and abbreviations.

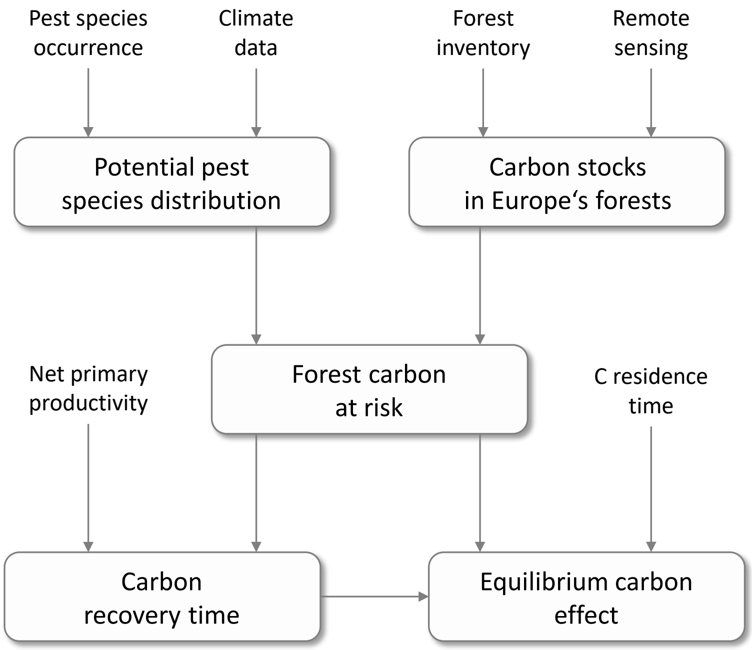

Supplementary Figure 9: Analytical framework for quantifying the potential carbon cycle impacts of invasive alien pest species in Europe’s forests.

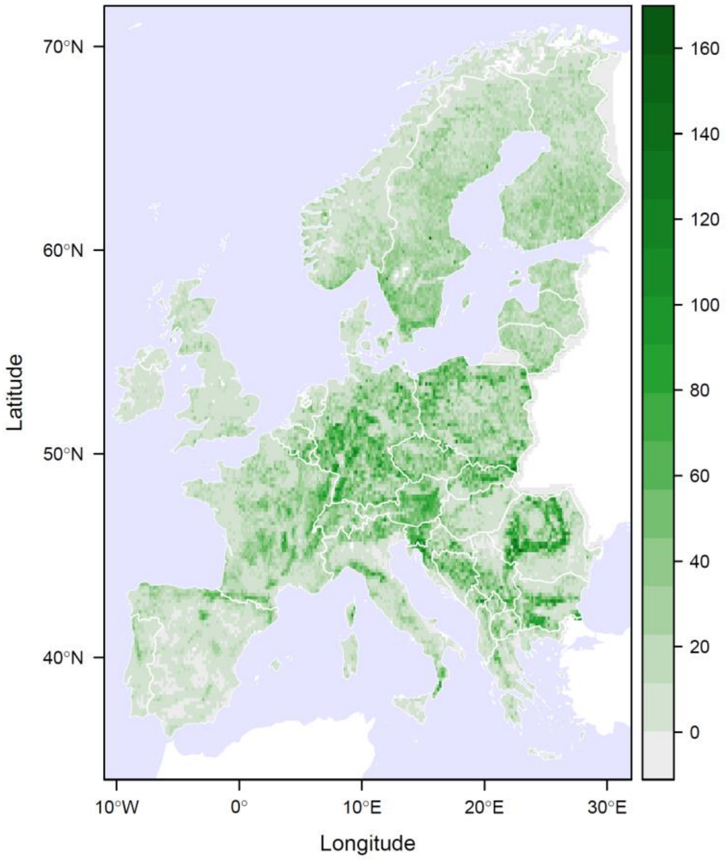

Supplementary Figure 10: Current live tree carbon stocks in Europe's forests (Mg C ha^{-1}) at a spatial grain of 10 arc minutes. Source: [20].

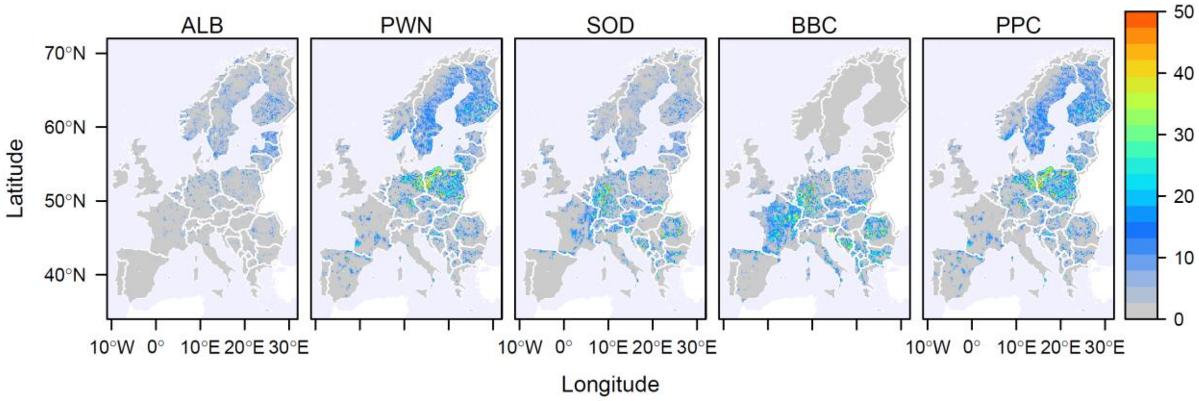

Supplementary Figure 11: Live tree C in the host tree species of the five alien pest species (Mg C ha^{-1}).

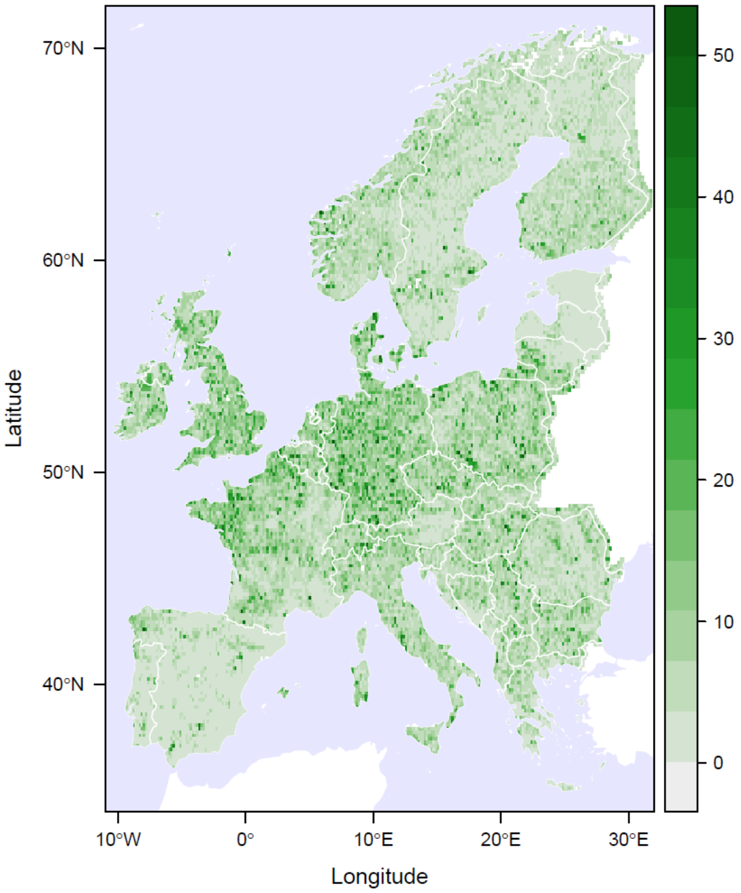

Supplementary Figure 12: Inventory-based within-grid-cell standard deviation (Mg C ha^{-1}) of live tree C in Europe's forests. Spatial grain: 10 arc minutes. Source: [20].

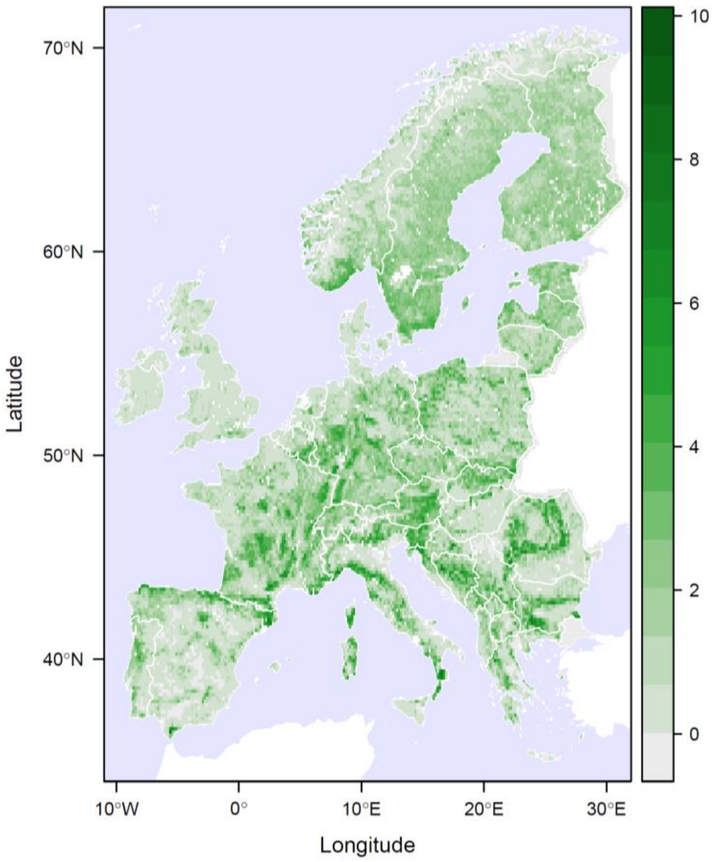

Supplementary Figure 13: Current NPP (Mg C ha^{-1} yr^{-1}) in Europe's forests at a spatial grain of 10 arc minutes. Source: [21].

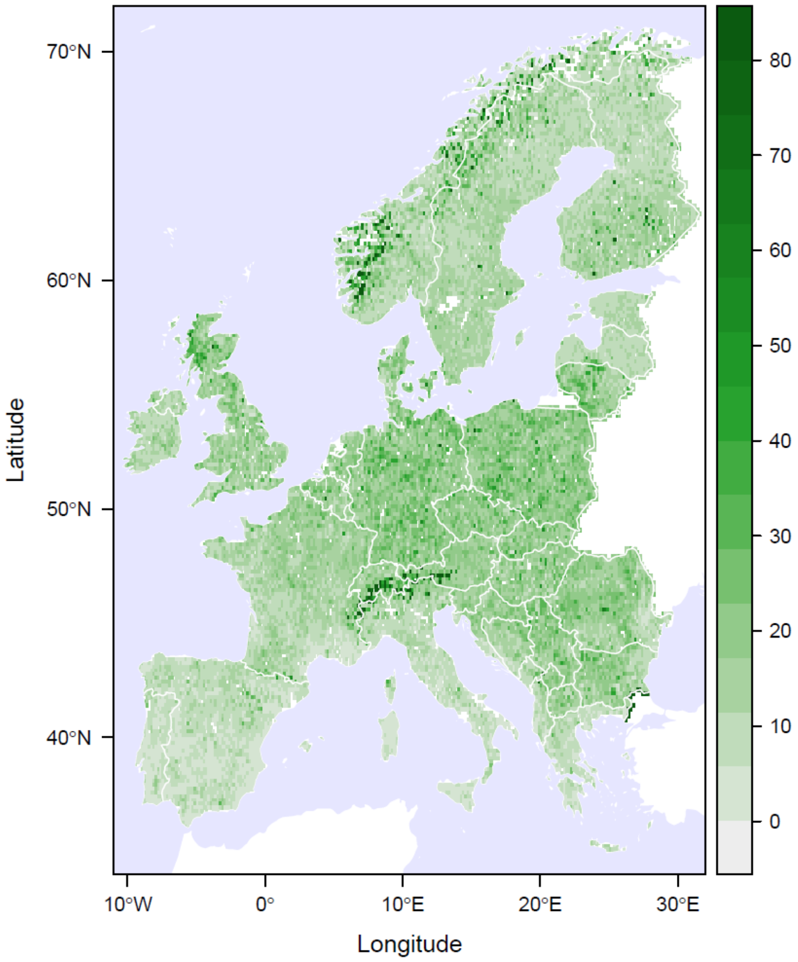

Supplementary Figure 14: Live C residence time (τ_l) in Europe's forests.

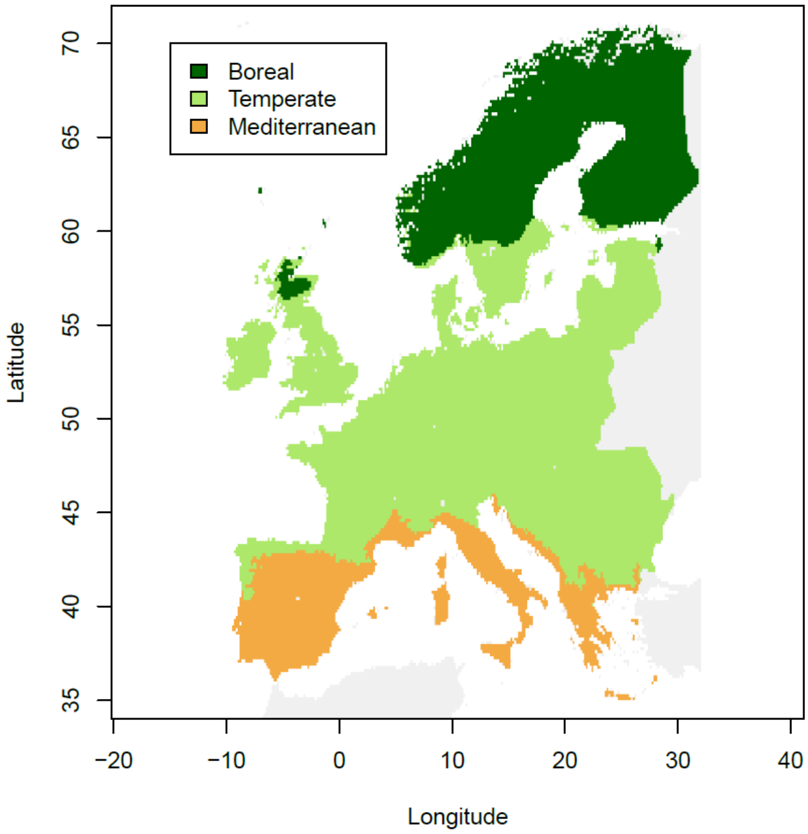

Supplementary Figure 15: Biomes of Europe [22].

Supplementary Table 1: Models and modelling groups that provided data on future climate scenarios via the CORDEX portal. RCM = regional climate model. ΔT = delta of mean annual temperature (2030-2080) relative to current climate. ΔP = rate of mean annual precipitation change (2030-2080) relative to current climate.

Institute	RCM name	Resolution	Driving model	Global Emission Scenario	ΔT (°C)	ΔP (%)
SMHI	RCA4	0.11 deg	EC-EARTH	rcp26	+1.1	+2.1
SMHI	RCA4	0.11 deg	CNRM-CM5	rcp45	+ 1.4	+5.7
DMI	HIRHAM5	0.11 deg	EC-EARTH	rcp85	+ 2.4	+5.0

SMHI: Swedish Meteorological and Hydrological Institute
DMI: Danish Meteorological Institute

Supplementary Table 2: Climatically suitable area for invasive alien pests under different climate scenarios and assumptions of model aggregation. lib: liberal model aggregation; cons: consensus predictor. Values are in Mill. km^2 land area.

Pest	Model assumption	Current climate (1950 – 2000)	RCP2.6 (2030 – 2080)	RCP4.5 (2030 – 2080)	RCP8.5 (2030 – 2080)
ALB	lib	3.719	3.686	3.980	4.069
	cons	3.169	2.892	3.260	2.848
PWN	lib	3.727	3.857	4.120	4.460
	cons	1.112	1.496	1.727	2.230
SOD	lib	1.231	1.828	1.558	1.841
	cons	0.943	1.348	1.186	1.394
BBC	lib	0.551	0.814	0.667	0.848
	cons	0.450	0.599	0.469	0.458
PPC	lib	2.455	3.489	3.469	3.649
	cons	1.942	2.848	2.907	3.135

Supplementary Table 3: Management and climate effect on live tree carbon at risk (Tg C). All: upper bound of C at risk from all five forest pest species. See Table 1 for species abbreviations.

Species	Pest management	Current climate (1950 – 2000)	RCP2.6 (2030– 2080)	RCP4.5 (2030 – 2080)	RCP8.5 (2030 –2080)
ALB	unmanaged	387.448	298.809	376.369	333.242
	managed	96.862	74.702	94.092	83.310
PWN	unmanaged	280.927	487.630	596.229	762.666
	managed	66.100	114.736	140.289	179.451
SOD	unmanaged	8.234	31.275	24.453	35.078
	managed	3.743	14.216	11.115	15.944
BBC	unmanaged	2.825	8.648	4.629	6.156
	managed	0.942	2.883	1.543	2.052
PPC	unmanaged	11.573	34.329	38.627	40.693
	managed	3.858	11.443	12.876	13.564
All	unmanaged	686.358	851.482	1026.899	1162.526
	managed	170.43	217.333	256.894	291.797

Supplementary Table 4: Climate and management effect on carbon recovery time (years) for all five pest species taken together.

Climate scenario	Pest management	
	with	without
Current climate (1950 – 2000)	17.3	29.9
RCP2.6 (2030– 2080)	18.2	31.9
RCP4.5 (2030 – 2080)	19.3	33.6
RCP8.5 (2030 –2080)	20.2	34.8

Supplementary Table 5: Effects of successful pest management.

Species	Time from infection to death (years)	Mortality unmanaged (mean and range)	Mortality managed (mean and range)	Comments	Sources
ALB (*Anoplophora glabripennis*)	5	80% (70-90%)	20% (10-30%)	Identification of infested trees using sniffer dogs, removal of infested trees, quarantining infested areas	2,6,23–25
PWN (*Bursaphelenchus xylophilus*)	1	85% (80-90%)	20% (10-30%)	Quarantining infested areas, removal of infested trees, controlling the vector species (*Monochamus* ssp.)	2,6,26,27
SOD (*Phytophthora ramorum*)	10	5.5% (1-10%)	2.5% (0-5%)	Removal of infested trees, quarantining infested areas to limit spread	2,6,28,29
BBC (*Phytophthora kernoviae*)	5	3% (1-5%)	1% (0-2%)	Removal of infested plants, quarantining infested areas, controlling the main host species (*Rhododendron* ssp.)	2,6,29
PPC (*Fusarium circinatum*)	5	3% (1-5%)	1% (0-2%)	Removal of infested trees, quarantining infested areas to limit spread	2,6,15,19,30

Supplementary Table 6: Equilibrium C cycle effect (Tg C). Values are derived from Eq. 4 and are comparable to the assessment for the current disturbance regime in Europe's forests presented by Seidl et al. [31]. All: upper bound of C at risk from all five species. See Table 1 for species abbreviations.

Pest	Treatment	Current climate (1950 – 2000)	RCP2.6 (2030 – 2080)	RCP4.5 (2030 – 2080)	RCP8.5 (2030 – 2080)
ALB	unmanaged	381.1	322.6	386.1	354.0
	managed	246.0	218.5	252.0	234.1
PWN	unmanaged	230.2	349.3	431.5	533.0
	managed	188.4	230.8	291.2	315.9
SOD	unmanaged	13.3	53.3	44.4	60.9
	managed	9.0	40.2	32.7	45.2
BBC	unmanaged	10.0	32.3	17.4	22.7
	managed	5.7	22.3	11.7	16.0
PPC	unmanaged	23.1	74.5	83.4	91.1
	managed	10.4	40.6	46.5	48.0
All	unmanaged	523.1	613.3	701.8	786.3
	managed	308.7	363.0	392.6	430.0

Supplementary Table 7: Response of the mean recovery time to potential changes in forest composition and functioning. The mean recovery time with default parameters was 34 years, and responses are reported as percent changes relative to the imposed changes in the focal variable.

Variable	Imposed change (%)	Response (%)
Carbon stocks	+10	+10.1
	+25	+25.1
NPP	-15	+17.8
	+15	-13.0
C share of host tree species	-5	-5.9
	+5	+15.8

Supplementary Table 8: Evaluation of the distribution models for the five forest pest species (see Table 1 for abbreviations). For each modelling approach the mean true skill statistic (TSS) over all replicates as well as the percentage of model replicates that have a TSS < 0.5 is reported. GLM: Generalized Linear Models, GAM: General Additive Models, RF: Random Forest, BRT: Boosted Regression Trees.

Species	GLM		GAM		RF		BRT	
	Mean TSS	%TSS<0.5	Mean TSS	%TSS<0.5	Mean TSS	%TSS<0.5	Mean TSS	%TSS<0.5
ALB	0.641	0.0	0.734	0.0	0.807	0.0	0.818	0.0
PWN	0.603	33.3	0.731	0.0	0.784	0.0	0.795	0.0
SOD	0.880	0.0	0.905	0.0	0.932	0.0	0.948	0.0
BBC	0.967	0.0	0.969	0.0	0.983	0.0	0.972	0.0
PPC	0.786	0.0	0.804	0.0	0.846	0.0	0.842	0.0

Supplementary Table 9: Response of the equilibrium C cycle effect of invasive pests to changes in the live C residence time and salvage fraction. The default salvage fraction was 1.0, and the default live C residence time varies across Europe (Supplementary Fig. 14). Responses are reported as percent changes relative to the imposed changes in the focal variable.

Variable	Imposed change (%)	Response (%)			
		Current climate (1950 – 2000)	RCP2.6 (2030– 2080)	RCP4.5 (2030 – 2080)	RCP8.5 (2030 –2080)
Live C residence time (τ_l)	+10	+9.4	+9.4	+9.3	+9.3
	-10	-9.5	-9.5	-9.4	-9.4
Salvage fraction	-20	-13.1	-13.3	-13.5	-13.8
	-34	-22.3	-22.5	-22.9	-23.5

Supplementary references

1. Ayberk, H., Ozdikmen, H. & Cebeci, H. A serious pest alert for Turkey: a newly introduced invasive longhorned beetle, Anoplophora glabripennis (Cerambycidae: Lamiinae). *Florida Entomol.* **97,** 1852–1855 (2014).
2. CABI. *Invasive Species Compendium*. (CAB International, 2017).
3. DAISIE. Delivering Alien Invasive Species Inventories for Europe. (2017). Available at: http://www.europe-aliens.org. (Accessed: 3rd April 2017)
4. Hölling, D. Der Asiatische Laubholzbockkäfer in Europa. *waldwissen.net* (2015). Available at: http://www.waldwissen.net. (Accessed: 3rd April 2017)
5. Liebhold, A. & Blackburn, L. Alien Forest Pest Explorer. *USDA Forest Service, Northern Research Station* (2016). Available at: https://www.nrs.fs.fed.us/tools/afpe/. (Accessed: 24th January 2017)
6. EPPO. *EPPO Global Database*. (European and Mediterranean Plant Protection Organization, 2017).
7. Garbelotto, M., Barbosa, D., Mehl, H. & Rizzo, D. M. First Report of the NA2 Lineage of *Phytophthora ramorum* from an Ornamental Rhododendron in the Interior of California. *Plant Dis.* **98,** 849–849 (2014).
8. GBIF. Global Biodiversity Information Facility. (2017). Available at: http://www.gbif.org/. (Accessed: 3rd April 2017)
9. King, K. M., Harris, A. R. & Webber, J. F. *In planta* detection used to define the distribution of the European lineages of *Phytophthora ramorum* on larch (*Larix*) in the UK. *Plant Pathol.* **64,** 1168–1175 (2015).
10. Olsen, L., Choffnes, E. R., Relman, D. A. & Pray, L. *Fungal diseases: An emerging threat to human, animal and plant health: Workshop summary*. (The National Academies Press, 2011).
11. Peterson, E., Hansen, E. & Hulbert, J. Source or sink? The role of soil and water borne inoculum in the dispersal of Phytophthora ramorum in Oregon tanoak

forests. *For. Ecol. Manage.* **322,** 48–57 (2014).

12. Webber, J. *Persistence of P. kernoviae and P. ramorum on infested sites: impact on disease management. 3rd International Phytophthora Workshop* (2008).

13. Britz, H., Couhnho, T. A., Gordon, T. R. & Wingfield, M. J. Characterisation of the pitch canker fungus, Fusarium circinatum, from Mexico. *South African J. Bot.* **67,** 609–614 (2001).

14. Correll, J. C. *et al.* Pitch Canker Disease in California: Pathogenicity, distribution, and canker development on Monterey pine (Pinus radiata). *Plant Disease* **75,** 676–682 (1991).

15. EFSA Panel on Plant Health. Risk assessment of Gibberella circinata for the EU territory and identification and evaluation of risk management options. *EFSA J.* **8,** 1620 (2010).

16. Pfenning, L. H. *et al.* First report and characterization of Fusarium circinatum, the causal agent of pitch canker in Brazil. *Trop. Plant Pathol.* **39,** 210–216 (2014).

17. Steenkamp, E. T., Rodas, C. A., Kvas, M. & Wingfield, M. J. Fusarium circinatum and pitch canker of Pinus in Colombia. *Australas. Plant Pathol.* **41,** 483–491 (2012).

18. Wingfield, M. J., Jacobs, A., Coutinho, T. A., Ahumada, R. & Wingfield, B. D. First report of the pitch canker fungus, Fusarium circinatum, on pines in Chile. *Plant Pathol.* **51,** 397–397 (2002).

19. Wingfield, M. J. *et al.* Pitch canker caused by *Fusarium circinatum* – a growing threat to pine plantations and forests worldwide. *Australas. Plant Pathol.* **37,** 319 (2008).

20. Moreno, A., Neumann, M. & Hasenauer, H. Forest structures across Europe. *Geosci. Data J.* **4,** 17–28 (2017).

21. Neumann, M. *et al.* Creating a regional MODIS satellite-driven net primary production dataset for European forests. *Remote Sens.* **8,** 554 (2016).

22. FAO. *Global ecological zones for FAO forest reporting: 2010 update*. (Forest Resources Assessment Working Paper 179, Food and Agricultural Organization of the United Nations, 2012).

23. Hu, J., Angeli, S., Schuetz, S., Luo, Y. & Hajek, A. E. Ecology and management of exotic and endemic Asian longhorned beetle Anoplophora glabripennis. *Agric. For. Entomol.* **11,** 359–375 (2009).

24. Smith, M. T., Turgeon, J. J., De Groot, P. & Gasman, B. Asian Longhorned Beetle Anoplophora glabripennis (Motschulsky): Lesson learned and opportunities to improve the process of eradication and management. *Am. Entomol.* **55,** 21–25 (2009).

25. EPPO. PM 9/15 (1) Anoplophora glabripennis: Procedures for official control. *EPPO Bull.* **43,** 510–517 (2013).

26. Sousa, E., Rodrigues, J. M., Bonifácio, L. F., Naves, P. M. & Rodrigues, A. Management and control of the Pine Wood Nematode , Bursaphelenchus xylophilus, in Portugal. in *Nematodes: Morphology, functions and management strategies* (eds. Boeri, F. & Chung, J. A.) 1–21 (Nova Science Publishers, 2011).

27. Sousa, E., Naves, P. & Vieira, M. Prevention of pine wilt disease induced by Bursaphelenchus xylophilus and Monochamus galloprovincialis by trunk injection of emamectin benzoate. *Phytoparasitica* **41,** 143–148 (2013).

28. Scottish Government. *Strategy for Phytophthora ramorum in Scotland for 2015 - 2017.* (Scottish Government, 2015).

29. Forestry Commission. Top tree diseases. (2016). Available at: https://www.forestry.gov.uk/forestry/BEEH-9XLGXD. (Accessed: 6th April 2017)

30. Schröder, T. New in Europe: the quarantine fungus Fusarium circinatum – situation, risk analysis, and quarantine measures. *Jahrb. der Baumpfl.* **2007,** 321–326 (2007).

31. Seidl, R., Schelhaas, M.-J., Rammer, W. & Verkerk, P. J. Increasing forest

disturbances in Europe and their impact on carbon storage. *Nat. Clim. Chang.* **4,** 806–810 (2014).
